# Association of Glucagon‐Like Peptide‐1 Receptor Agonists and Suicidality: A Systematic Review

**DOI:** 10.1111/obr.70120

**Published:** 2026-03-06

**Authors:** Hezekiah C. T. Au, Yang Jing Zheng, Gia Han Le, Sabrina Wong, Kayla M. Teopiz, Angela T. H. Kwan, Joshua D. Rosenblat, Rodrigo B. Mansur, Hayun Choi, Roger S. McIntyre

**Affiliations:** ^1^ Joint Department of Medical Imaging University of Toronto Toronto Ontario Canada; ^2^ Institute of Medical Science University of Toronto Toronto Ontario Canada; ^3^ Brain and Cognition Discovery Foundation Toronto Ontario Canada; ^4^ Mood Disorder Psychopharmacology Unit University Health Network Toronto Ontario Canada; ^5^ Department of Pharmacology and Toxicology University of Toronto Toronto Ontario Canada; ^6^ Faculty of Medicine University of Ottawa Ottawa Ontario Canada; ^7^ Department of Psychiatry University of Toronto Ontario Canada; ^8^ Department of Psychiatry Veteran Health Service Medical Center Seoul Republic of Korea

**Keywords:** GLP‐1 receptor agonists, glucagon‐like peptide, obesity, suicidality

## Abstract

**Introduction:**

Increased risk of suicidality has been reported in association with glucagon‐like peptide receptor agonist (GLP‐1 RA) prescription. Herein, we conducted a comprehensive review evaluating reports of GLP‐1 RA prescription and suicidality.

**Methods:**

Relevant articles were retrieved from OVID (Medline, EMBASE, AMED, PsycINFO, JBI EBP Database), PubMed, and Web of Science from inception to May 20, 2024. Primary research examining the association between GLP‐1 RAs (i.e., dulaglutide, exenatide, liraglutide, lixisenatide, semaglutide, tirzepatide) and suicidality were included for analysis.

**Results:**

Our findings indicate liraglutide (reported odds ratio [ROR] = 3.26, 95% CI = 2.53, 4.22) and semaglutide (ROR = 1.73; 95% CI = 0.30, 0.80) are significantly associated with a greater odds ratio of reported suicidal ideation. Similarly, tirzepatide was associated with greater odds of reported suicidal ideation; however, this was nonsignificant (ROR = 1.49; 95% CI = −0.41, 1.21). Similarly, semaglutide (ROR = 8.81; 95% CI = 3.69, 21.04) and liraglutide (ROR = 3.74; 95% CI = 1.23, 11.38) are also associated with a greater odds ratio of reported suicidal depression. No significant association between other GLP‐1 RAs and suicidality was observed.

**Discussion:**

Reports of aspects of suicidality and exposure to select GLP‐1 RAs exist; notwithstanding, no causality between GLP‐1 RA exposure and suicidality is apparent.

## 1 Introduction

Glucagon‐like peptide‐1 receptor agonists (GLP‐1 RAs) are commonly prescribed in the treatment of type 2 diabetes mellitus (T2DM) [1]. In addition, GLP‐1 RAs have been reported to benefit aspects of psychopathology including depressive symptoms, cognitive impairment in persons with major neurocognitive disorders, and psychotropic drug‐related weight gain [2, 3, 4, 5, 6].

Suicidality comprised three interrelated aspects: suicidal ideation (i.e., thoughts of ending own life), suicidal behavior and completed suicide [7, 8]. In addition, “suicidal depression” is a category within the Food and Drug Administration (FDA) Adverse Event Reporting System (FAERS) that captures reports of suicidality, characterized by depression with suicidal thoughts [9]. Recently, the European Medicines Agency (EMA) and the US FDA issued public statements that, although an association between GLP‐1 RAs and suicidality may exist, no causality can be established [10, 11, 12].

Herein, we attempt to synthesize reports documenting an association between GLP‐1 RAs and aspects of suicidality. Additionally, we briefly examine the effects of GLP‐1 receptor agonism and depression to understand potential effects that subserve the relation between GLP‐1 RA administration and suicidality. The overarching aim is to provide a repository of extant data on the topic to guide further research.

## 2 Methods

### 2.1 Search Strategy

This study was conducted using the 2020 PRISMA (Preferred Reporting Items for Systematic Reviews and Meta‐Analyses) [13]. A systematic search for relevant articles was conducted on Web of Science, OVID (Medline, EMBASE, AMED, PsycINFO, JBI EBP), and PubMed from database inception to May 20, 2024. The search string applied for the search included: (“GLP‐1” OR “Glucagon‐Like Peptide‐1” OR “Glucagon‐Like Peptide 1” OR “GLP‐1 Agonist” OR “Glucagon‐Like Peptide‐1 Agonist” OR “Glucagon‐Like Peptide 1 Agonist” OR “Semaglutide” OR “Ozempic” OR “Rybelsus” OR “Wegovy” OR “Dulaglutide” OR “Trulicity” OR “Exenatide” OR “Byetta” OR “Bydureon” OR “Liraglutide” OR “Lixisenatide”) AND (“Suicide attempts” OR “Suicidal ideation” OR “Suicidal behavior” OP “Suicidal behav*” OR “Suicid*”). A separate search was completed on Google Scholar and from reference lists to ensure all relevant articles were captured.

### 2.2 Study Selection and Inclusion Criteria

The Covidence platform was used to screen articles obtained from the systematic search, wherein duplicate articles were removed [14]. Two independent reviewers (H.A. and Y.J.Z.) screened the titles and abstracts based on the inclusion and exclusion criteria (Table 1). Primary articles were retrieved by full‐text screening by two reviewers (H.A. and Y.J.Z.) if they reported on suicidal ideation or suicidality as a result of GLP‐1RA prescription (refer to Table 1 for detailed eligibility criteria).

**TABLE 1:** Eligibility criteria.

Inclusion criteria	A primary study Measurement of suicide attempt or suicidal ideation Full‐text article available online English language
Exclusion criteria	Nonprimary or secondary research (i.e., literature reviews, systematic reviews, meta‐analyses, posters, abstracts, guidelines, protocols, and theses) Participants must not be diagnosed with comorbidities or mixed diagnoses between different disorders Case studies Animal studies Reports an association without statistics Full text is not available

### 2.3 Data Extraction

Relevant information was organized and obtained from included studies using the piloted data extraction template. Information to be extracted was established a priori, including (1) author, (2) study type, (3) sample size, (4) diagnoses, and (5) outcomes of interest. Two reviewers (H.A. and Y.J.Z.) conducted data extraction, wherein all conflicts were resolved via discussion. Outcomes of interest examined instances of suicidality associated with GLP‐1RA prescription.

### 2.4 Quality Assessments

Quality assessment of observational cohort studies was conducted using the Quality Assessment for Observational Cohort and Cross‐Sectional Studies from the National Institutes of Health (NIH) [15, 16]. Similarly, risk‐of‐bias assessments for randomized controlled trials and post hoc analyses were conducted using the Quality Assessment of Controlled Intervention Studies from the NIH by two independent reviewers (H.A. and Y.J.Z.), wherein all conflicts were resolved through discussion [15, 16]. Further information on the methodological quality assessments has been listed in the Supporting Information (Table S1, Table S2).

## 3 Results

### 3.1 Search Results

**FIGURE 1:**
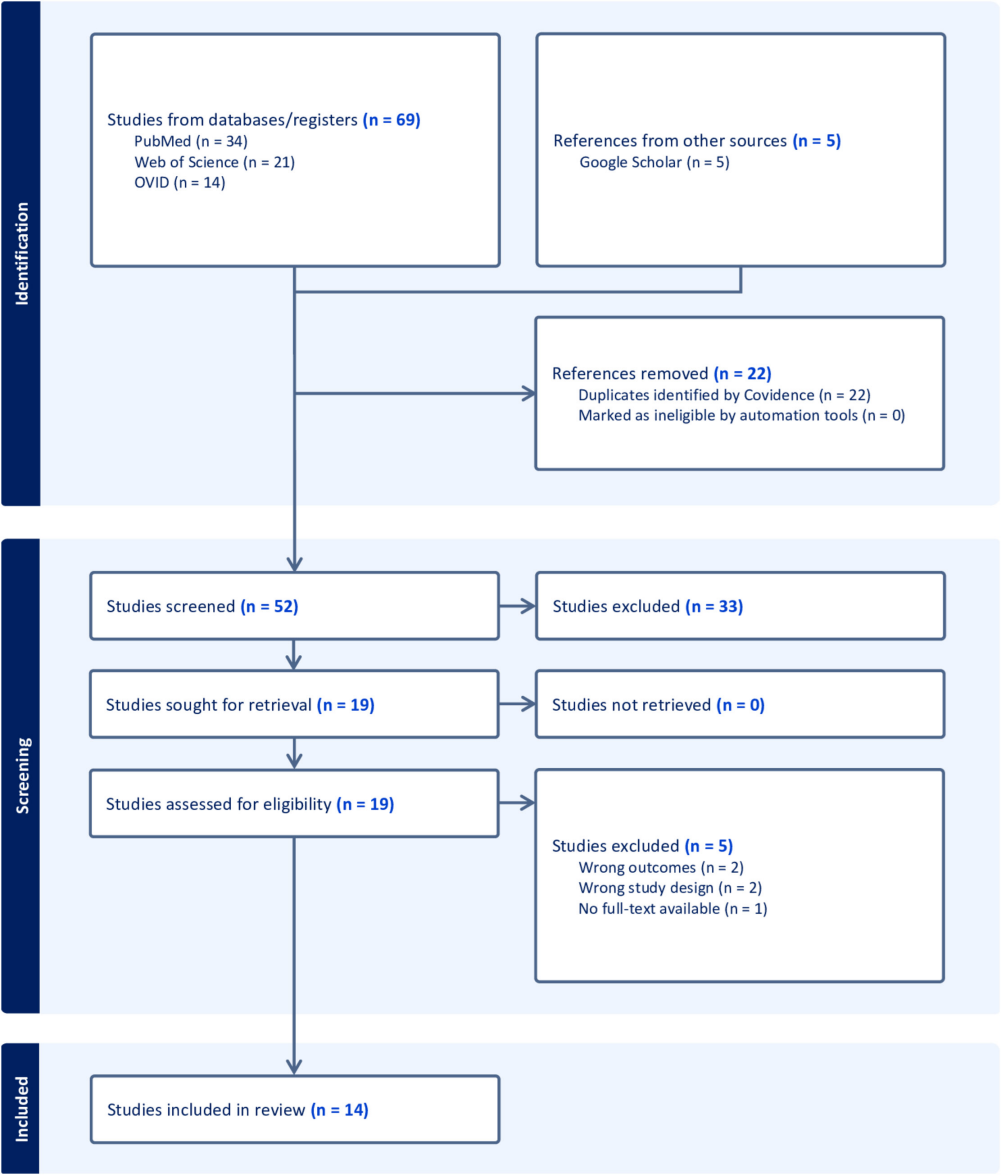
PRISMA flow diagram of literature search (Covidence, 2024).

A systematic database search yielded 69 results. Further manual searches on Google Scholar yielded five articles. Twenty‐two duplicates were removed through Covidence, wherein abstract and title screening proceeded for the remaining 52 articles. Nineteen relevant full‐text articles were retrieved and screened based on the eligibility criteria (Table 1). Five studies were excluded as a result of wrong outcomes (*n* = 2), wrong study design (*n* = 2), or no full‐text available (*n* = 1) (Figure 1). Fourteen studies that met the eligibility criteria were included in this systematic review. Study‐specific results and experimental designs have been listed in Table 2.

**TABLE 2:** Characteristics of Studies Examining Association Between GLP‐1 and Suicidality.

Author(s)	Study type	Sample size	Diagnoses	Outcome(s) of interest
Suicidal ideation				
Guirguis et al. (2024) [17]	Pharmacovigilance analysis	209,354 total participants 9610 participants prescribed with albiglutide 58,207 participants prescribed with dulaglutide 77,019 participants prescribed with exenatide 35,774 participants prescribed with liraglutide 166 participants prescribed with lixisenatide 18,923 participants prescribed with semaglutide 9655 participants prescribed with tirzepatide	N/A	Albiglutide: Out of all 9610 ADR cases, three cases of suicide and self‐injury related cases were reported, including: Three counts of suicidal ideation Dulaglutide: Out of all 58,207 ADR cases, 59 cases of suicide and self‐injury related cases were reported, including: 34 cases of suicidal ideation 20 cases of suicidal attempts 3 counts of completed suicide 3 counts of suicidal behavior 2 counts of suicidal depression Exenatide: Out of all 77,019 ADR cases, 96 cases of suicide and self‐injury related cases were reported, including: 62 counts of suicidal ideation 18 counts of suicidal attempts 2 counts of suicidal behavior 2 counts of self‐injurious ideation Liraglutide: Out of all 35,774 ADR cases, 143 cases of self‐injurious or suicidal behavior were reported, including: 71 counts of suicidal ideation 28 counts of suicidal attempts 25 counts of completed suicide were reported 5 counts of suspected suicide 3 counts of suicidal behavior 2 counts of suicidal depression 4 counts of self‐injurious ideation 5 counts of intentional self‐injury Lixisenatide: Out of all 166 ADR cases, no counts of suicide and self‐injury related cases were reported. Semaglutide: Out of all 18,923 ADR cases, 76 cases of self‐injurious or suicidal behavior were reported, including: 60 counts of suicidal ideation 7 counts of suicidal attempts 7 counts of suicidal depression 1 count of self‐injurious ideation 1 count of intentional self‐injury Tirzepatide: Out of 9655 ADR cases, six cases of suicide and self‐injury related cases were reported, including: Six counts of suicidal ideation. General observations: 383 cases of suicidal or self‐injurious ideation were reported out of 209,354 total reports of adverse drug reactions (ADRs) for all seven GLP‐1 RAs. Reported odds ratio (OR) for all seven RAs were: liraglutide (*n* = 143; reported odds ratio (ROR) = 1.97; 95% confidence interval [CI] = 0.89, 0.47), exenatide (*n* = 96; ROR = 0.81; 95% CI = 0.03, −0.44), semaglutide (*n* = 76; ROR = 1.73; 95% CI = 0.80, 0.30), dulaglutide (*n* = 59; ROR = 0.77; 95% CI = 0.01, −0.54), tirzepatide (*n* = 6; ROR = 1.49; 95% CI = 1.21, −0.41), albiglutide (*n* = 3; ROR = 0.05; 95% CI = −1.94, −4.22), and lixisenatide (*n* = 0; ROR = 0). Pharmacovigilance disproportionality analyses using ROR identified that GLP‐1 RAs were associated with suicidal and self‐injurious ideation, in descending order of liraglutide, semaglutide, and tirzepatide. After unmasking analysis, individual drugs were implicated as the sole drug in selected ADRs for 271 cases, wherein liraglutide (*n* = 90; ROR = 1.64; 95% CI = 0.75, 0.24), exenatide (*n* = 67; ROR = 0.80; 95% CI = 0.05, −0.50), semaglutide (*n* = 61; ROR = 2.03; 95% CI = 0.99, 0.42), dulaglutide (*n* = 45; ROR = 0.84; 95% CI = 0.14, −0.50), tirzepatide (*n* = 5; ROR = 1.76; 95% CI = 1.45, −0.32), albiglutide (*n* = 2; ROR = 0.04; 95% CI = −1.75, −4.53), and lixisenatide (*n* = 0; ROR = 0) After unmasking analysis, pharmacovigilance disproportionality analyses showed that GLP‐1 RAs were associated with suicidal and self‐injurious ideation in descending order, wherein ROR values are all above 1.0: semaglutide, tirzepatide, and liraglutide.
McIntyre et al. (2023) [12]	Pharmacovigilance analysis	45,415 total participants 1,317 participants prescribed with semaglutide 2,189 participants prescribed with dulaglutide 4,274 participants prescribed with exenatide 1,546 participants prescribed with liraglutide 10 participants prescribed with lixisenatide 389 participants prescribed with tirzepatide 16,841 participants prescribed with insulin 20,241 participants prescribed with metformin	N/A	RORs comparing role of GLP‐1 RAs and metformin in suicidal ideation: semaglutide (ROR = 4.08; 95% CI = 3.17, 5.25; *p* ≤ 0.0001), dulaglutide (ROR = 1.03; 95% CI = 0.72, 1.46; *p* = 0.89), exenatide (ROR = 0.95; 95% CI = 0.72, 1.24; *p* = 0.68), liraglutide (ROR = 3.26; 95% CI = 2.53, 4.22; *p* ≤ 0.0001), lixisenatide (ROR 0), and tirzepatide (ROR = 1.67; 95% CI = 0.88, 3.15; *p* = 0.12), respectively. RORs comparing role of GLP‐1 RAs and metformin in depressive/suicidal: semaglutide (ROR = 8.81; 95% CI = 3.69, 21.04; *p* ≤ 0.0001), dulaglutide (ROR = 1.32; 95% CI = 0.30, 5.81; *p* = 0.71), exenatide (ROR = 2.37; 95% CI = 0.95, 5.86; *p* = 0.063), liraglutide (ROR = 3.74; 95% CI = 1.23, 11.38; *p* ≤ 0.05), lixisenatide (ROR 0), and tirzepatide (ROR 0), respectively. RORs comparing role of GLP‐1 RAs and metformin for suicidal behaviors: semaglutide (ROR = 0), dulaglutide (ROR = 1.42, 95% CI = 0.32, 6.30; *p* = 0.64), exenatide (ROR 0.73; 95% CI = 0.16, 3.22; *p* = 0.67), liraglutide (ROR = 3.02; 95% CI = 0.86, 10.61; *p* = 0.085), lixisenatide (ROR = 0), and tirzepatide (ROR = 0), respectively. RORs comparing role of GLP‐1 RAs and metformin for suicidal attempts: semaglutide (ROR = 0.048; 95% CI = 0.023, 0.10; *p* ≤ 0.0001), dulaglutide (ROR = 0.083; 95% CI = 0.054, 0.13; *p* ≤ 0.0001), exenatide (ROR = 0.038; 95% CI = 0.024, 0.061; *p* ≤ 0.0001), liraglutide (ROR = 0.17; 95% CI = 0.12, 0.25; *p* ≤ 0.0001), lixisenatide (ROR = 0), and tirzepatide (ROR = 0), respectively. RORs comparing role of GLP‐1 RAs and metformin for completed suicide: semaglutide (ROR = 0.0078; 95% CI = 0.0029, 0.021; *p* ≤ 0.001), dulaglutide (ROR = 0.0035; 95% CI = 0.0011, 0.011; *p* ≤ 0.0001), exenatide (ROR = 0.0030; 95% CI = [0.0013, 0.0072; *p* ≤ 0.0001), liraglutide (ROR = 0.044, 95% CI = 0.030, 0.065; *p* ≤ 0.001), lixisenatide (ROR = 0), and tirzepatide (ROR = 0), respectively Insulin: RORs comparing role of GLP‐1 RAs and insulin in suicidal ideation: semaglutide (ROR = 4.53; 95% CI = 3.49, 5.88; *p* ≤ 0.0001), dulaglutide (ROR = 1.14; 95% CI = 0.80, 1.63; *p* = 0.48), exenatide (ROR 1.05; 95% CI = 0.79, 1.39; *p* = 0.74), liraglutide (ROR = 3.62; 95% CI = 2.78, 4.72; *p* ≤ 0.0001), lixisenatide (ROR = 0), and tirzepatide (ROR = 1.85; 95% CI = 0.97, 3.51; *p* = 0.06), respectively. RORs comparing role of GLP‐1 RAs and insulin in depressive/suicidal: semaglutide (ROR = 9.35, 95% CI = 3.75, 23.29; *p* ≤ 0.0001), dulaglutide (ROR = 1.40; 95% CI = 0.31, 6.32; *p* = 0.66), exenatide (ROR = 2.51; 95% CI = 0.97, 6.48; *p* = 0.06), liraglutide (ROR = 3.97; 95% CI = 1.26, 12.48; *p* ≤ 0.05), lixisenatide (ROR = 0), and tirzepatide (ROR = 0), respectively. RORs comparing role of GLP‐1 RAs and insulin for suicidal behaviors: semaglutide (ROR = 0), dulaglutide (ROR = 1.10; 95% CI = 0.25, 4.84; *p* = 0.90), exenatide (ROR = 0.56; 95% CI = 0.13, 2.48; *p* = 0.45), liraglutide (ROR = 2.34; 95% CI = 0.67, 8.14; *p* = 0.18), lixisenatide (ROR = 0), and tirzepatide (ROR = 0), respectively. RORs comparing role of GLP‐1 RAs and insulin for suicide attempts: ROR = 0.097; 95% CI = 0.046, 0.21; *p* ≤ 0.0001), dulaglutide (ROR = 0.17; 95% CI = 0.11, 0.26; *p* ≤ 0.0001), exenatide (ROR = 0.077; 95% CI = 0.048, 0.12; *p* ≤ 0.0001), liraglutide (ROR = 0.35; 95% CI = 0.24, 0.51; *p* ≤ 0.0001), lixisenatide (ROR = 0), and tirzepatide (ROR = 0), respectively. RORs comparing role of GLP‐1 RAs and insulin for completed suicide: semaglutide (ROR = 0.16; 95% CI = 0.059, 0.43; *p* ≤ 0.001), dulaglutide (ROR = 0.071; 95% CI = 0.023, 0.22; *p* ≤ 0.0001), exenatide (ROR = 0.061; 95% CI = 0.025, 0.15; *p* ≤ 0.0001), liraglutide (ROR = 0.89; 95% CI = 0.59, 1.33; *p* = 0.57), lixisenatide (ROR = 0), and tirzepatide (ROR = 0)
O'Neil et al. (2017) [18]	Post hoc of randomized controlled trial	5,325 total participants 3384 participants prescribed with liraglutide 1,941 participants prescribed with placebo	Obesity	Participants were prescribed with 3.0 mg liraglutide daily, ramping up from 0.6 mg and increasing by 0.6 mg per week. Nine of 3384 individuals in the liraglutide group reported suicidal ideation or behavior. One individual did not recover from adverse events, but had history of suicidal attempt and should not have been included in the study. A total of 11 individuals of 5,325 individuals reported suicidal ideation or behavior, wherein all except one in each treatment group reported a past history of psychiatric disorder or life stressors. 34 individuals (1.0%) receiving liraglutide vs. 19 individuals (1.0%) receiving placebo reported suicidal ideation based on the C‐SSRS. Liraglutide was associated with decreases in mean PHQ‐9 scores following treatment (1.8 ± 2.7 vs. 1.9 ± 2.7), with a treatment difference of −0.02 (95% CI = −0.17, 0.12).
Shapiro et al. (2025) [19]	Retrospective cohort study	270,110 total participants 36,082 participants prescribed with GLP‐1 RAs	N/A	Prescription of GLP‐1 RAs were not significantly associated with differences in hazard ratio (HR) for suicidal ideations (HR = 0.84; 95% CI = 0.65, 1.08) and suicide (HR = 0.14; 95% CI = 0.02, 1.31).
Wadden et al. (2023) [20]	Randomized controlled trial	579 total participants 287 participants prescribed with tirzepatide 292 participants prescribed with placebo	Obesity	Individuals were administered with either 10 or 15 mg weekly for 72 weeks, increasing from 2.5 mg weekly in increments of 2.5 mg. Out of 287 participants prescribed with tirzepatide, one participant (0.3%) reported severe or serious MDD/suicidal behavior and ideation. Out of 292 participants prescribed with placebo, no reports of severe or serious MDD/suicidal behavior and ideation.
Wadden et al. (2024) [21]	Post hoc of randomized controlled trials	3,681 total participants 2,218 participants prescribed with semaglutide	Obesity T2DM	Participants were prescribed 2.4 mg semaglutide, once weekly for 68 weeks (STEP 1, 2, 3) or 104 weeks (STEP 5). Throughout STEP 1, 2, and 3, eight participants (0.4%) prescribed with semaglutide reported suicidal ideations, in comparison to seven participants (0.6%) prescribed with placebo. In STEP 5, one participant (0.7%) prescribed with semaglutide reported suicidal ideations, in comparison to two participants (1.4%) prescribed with placebo. Prescription of semaglutide was significantly associated with reduced maximum PHQ‐9 scores following 68 weeks (OR = 0.54; 95% CI = 0.37, 0.80; *p* = 0.002), but not following 104 weeks (OR = 0.76; 95% CI = 0.10, 5.80; *p* = 0.79) of semaglutide administration.
Wang et al. (2024) [22]	Retrospective cohort study	232,771 total participants 52,783 participants prescribed with semaglutide	Obesity	Significantly lower risk for incident suicidal ideation was observed in the semaglutide group than the non‐GLP1 RA antiobesity medication group (0.11% vs. 0.43%; HR = 0.27; 95% CI = 0.20–0.36). Among the semaglutide group (*n* = 52,783), no patient reported suicide attempts during the 6‐month follow‐up after semaglutide prescription. In comparison, 14 patients reported suicidal attempts in the non–GLP‐1 RA antiobesity medication group (*n* = 52,783) during the 6‐month follow‐up after medication prescription (*p* < 0.001). The semaglutide group was associated with significantly lower risk of recurrent experience of suicidal ideation (6.5% vs. 14.1%; HR = 0.44; 95% CI = 0.32, 0.60). Semaglutide was associated with a lower risk of incident suicidal ideation at 1‐year follow‐up (HR = 0.39; 95% CI = 0.28, 0.53). Associations were attenuated at 2‐year (HR = 0.53; 95% CI = 0.41, 0.67) and 3‐year follow‐up (HR = 0.58, 95% CI = 0.49, 0.72), but still significant.
Suicidal attempts				
Bezin et al. (2025) [23]	Retrospective cohort study	6,596 total participants 1,102 participants prescribed with GLP‐1 RAs	N/A	Administration of GLP‐1 RAs (dulaglutide, exenatide, liraglutide, semaglutide) in individuals admitted to the hospital was not associated with increased suicidal behaviors (OR = 0.62; 95% CI = 0.51, 0.75).
Chen et al. (2023) [24]	Pharmacovigilance analysis	288,138 participants reporting suicide/self‐injurious behavior 219,376 participants reporting adverse effects from GLP‐1RAs	N/A	Out of 288,138 reports of suicide and self‐injury, 534 cases were associated with GLP‐1RAs. Of which, the cases were categorized into: 50 cases of completed suicide 24 cases of suicidal depression 100 cases of intentional overdose 12 cases of intentional self‐injury 12 cases of deliberate poisoning 7 cases of self‐injurious ideation 8 cases of suicidal behavior 275 cases of suicidal ideation 103 cases of suicidal attempts 5 cases of suspected suicide Of which, 199 cases were associated with liraglutide, 134 cases associated with exenatide, 106 cases associated with semaglutide, 89 cases associated with dulaglutide, 3 cases associated with albiglutide, and 3 cases associated with lixisenatide. Stratified analysis by age showed that ROR for suicide and self‐injury with GLP‐1RAs were elevated in children (ROR = 2.50; 95% CI = 1.02, 6.13; *p* = 0.05).
Kelly et al. (2020) [25]	Randomized controlled trial	251 total participants 125 participants prescribed with liraglutide 126 participants prescribed with placebo	Obesity	Individuals were prescribed 3.0 mg liraglutide daily for 56 weeks, increasing from an initial 0.6 mg liraglutide daily. Suicide‐related events were reported in three participants in the liraglutide group. Site investigators deemed suicide events were unlikely to be related to trial treatment. One participant died by suicide. Two participants reported suicide attempts.
Nassar et al. (2024) [26]	Retrospective cohort study	745,985 total participants 373,041 participants prescribed with GLP‐1RAs 372,944 participants prescribed with DPP‐4i	T2DM	Out of 373,041 participants with T2DM treated with GLP‐1RAs, 106 counts of suicidal attempts were reported (28.41 per 100,000). Out of 372,944 participants with T2DM treated with DPP‐4is, 230 suicidal attempts were reported (61.67 per 100,000). The risk difference between two cohorts was significant (−33 per 100,000; 95% CI = −43, −24; *p* < 0.001) with an OR of 0.461 (95% CI = 0.366, 0.58). Out of 88,325 participants with a history of depression or suicide attempts and T2DM treated with GLP‐1 RAs, 68 counts of suicidal attempts were reported (76.98 per 100,000). Out of 88,623 participants with a history of depression or suicide attempts and T2D treated with DPP‐4i, 176 counts of suicidal attempts were reported (203.79 per 100,000). The risk difference between the two cohorts were significant (−127; 95% CI = −162, 92; *p* < 0.001) with an OR of 0.377 (95% CI = 0.285, 0.499).
Ruggiero et al. (2024) [27]	Pharmacovigilance analysis	41,236 total participants	N/A	Out of 41,236 safety reports, 230 cases reported at least one suicidal events, of which: 88 cases of suicidal events were associated with liraglutide 84 cases of suicidal events were associated with semaglutide 37 cases of suicidal events were associated with dulaglutide 16 cases of suicidal events were associated with exenatide 5 cases of suicidal events were associated with liraglutide/insulin degludec Probability of reporting suicidal events was higher in semaglutide than in dulaglutide (ROR = 2.05; 95% CI = 1.40, 3.01; *p* = 0.0002) or exenatide (ROR = 1.81; 95% CI = 1.08, 3.05; *p* = 0.0229). Probability for reporting suicidal events was higher in liraglutide than dulaglutide (ROR = 3.98; 95% CI = 2.73, 5.82; *p* < 0.0001) or exenatide (ROR = 3.52; 95% CI = 2.10, 5.92; *p* < 0.0001). Contrarily, probability of reporting suicidal events was lower in semaglutide than in liraglutide (ROR = 0.51; 95% CI = 0.38, 0.69; *p* < 0.0001). Liraglutide: Out of 90 cases of suicidal events, the events were categorized into: 5 counts of completed suicide 5 counts of suicidal depression 1 count of suicidal behavior 60 counts of suicidal ideation 16 counts of suicide attempt 3 counts of suspected suicide Semaglutide: Out of 86 counts of suicidal events, the events were categorized into: 3 counts of completed suicide 9 counts of suicidal depression 67 counts of suicidal ideation 7 counts of suicide attempt Dulaglutide: Out of 38 counts of suicidal events, the events were categorized into: 2 counts of completed suicide 2 counts of suicidal depression 2 counts of suicidal behavior 17 counts of suicidal ideation 15 counts of suicide attempts Exenatide: Out of 17 counts of suicidal events, the events were categorized into: 1 count of completed suicide 1 count of suicidal depression 2 counts of suicidal behavior 10 counts of suicidal ideation 3 counts of suicide attempt Liraglutide/degludec Out of five counts of suicidal events, the events were categorized into: Five counts of suicide attempts.
Tobaiqy and Elkout (2024) [28]	Pharmacovigilance analysis	31,444 total participants	N/A	Out of 31,444 ADR reports of adverse side effects, 13,956 were associated with semaglutide (44.4%), 16,748 were associated with liraglutide (53.3%), and 740 were associated with tirzepatide (2.3%). Suicide‐related adverse events: 102 adverse events were related to suicide, of which the events were categorized into: 73 counts of suicidal events 12 counts of suicidal attempts 10 counts of depression with suicidal ideation 4 counts of completed suicide 2 counts of suspected suicide 1 count of suicidal behavior Of the 102 events, 51 occurred with semaglutide, 48 occurred with liraglutide, and 4 were associated with tirzepatide.
Suicidal depression				
Blackman et al. (2016) [29]	Randomized controlled trial	359 total participants 176 participants prescribed with liraglutide 179 participants prescribed with placebo	Obesity	Suicidal depression was reported in one participant in the liraglutide group.

Abbreviations: C‐SSRS = Columbia‐Suicide Severity Rating Scale, N/A = not available, PHQ‐9 = Patient Health Questionnaire‐9, T2DM = type 2 diabetes mellitus.

### 3.2 Methodological Quality

All controlled intervention studies were described as double‐blinded, randomized trials and examined outcomes using validated measures. No clinical studies had a high risk of bias. The most common limitation was the lack of provided information regarding randomization and concealment of treatment allocation. Furthermore, the overall dropout rate due to treatment‐emergent adverse events was > 20% in some studies. The results from observational cohort studies provided clarity on study outcomes and population while incorporating a sufficient time frame to observe associations between exposure and outcomes. No clinical studies were identified as having a high risk of bias. Common limitations among included studies were missing reports of patient recruitment, omission of sample size justification, and a lack of adjustment for confounding variables. As a result of the retrospective nature of included studies, outcome assessors were not blinded to the exposure status of participants. Albeit the presence of limitations, the qualities of the results were not affected.

### 3.3 Association Between GLP‐1 RAs and Suicidality

#### 3.3.1 Association Between GLP‐1 RAs and Suicidal Ideation

To examine the association between GLP‐1 RAs and suicidal ideation, we identified 7 studies [12, 17, 18, 19, 21, 22, 27, 28]. In a post hoc analysis of a randomized clinical trial conducted by O'Neil et al. (2017) (*n* = 5,325), liraglutide was not associated with increased suicidal ideation based on assessments from the Columbia‐Suicide Severity Rating Scale (C‐SSRS), as a similar proportion of participants prescribed with liraglutide and healthy control reported suicidal ideation (1.0% vs. 1.0%) [18]. Contrastingly, a pharmacovigilance analysis conducted by McIntyre et al. [12] using data from the FAERS (*n* = 45,415) identified disproportionate reporting of suicidal ideation in liraglutide (reported odds ratio [ROR] = 3.26; 95% CI = 2.53, 4.22; *p* ≤ 0.0001) [12]. Similarly, Guirguis et al. (2024) identified an association between liraglutide and greater probability of suicidal ideation (*n* = 143; ROR = 1.97; 95% CI = 0.89, 0.47) [17]. This association is further reinforced by Ruggiero et al. (2024) (*n* = 41,236), wherein suicidal ideation was primarily reported with liraglutide prescription (60 of 90 suicidal events; 66.7%) [27]. Disproportionate analysis also indicated that a numerically higher rate of suicidal event reports were associated with liraglutide in comparison to dulaglutide (ROR = 3.98; 95% CI = 2.73, 5.82; *p* < 0.0001), exenatide (ROR = 3.52; 95% CI = 2.10, 5.92; *p* < 0.0001) [27]. Similarly, a numerically lower rate of suicidal ideation was observed in semaglutide when compared to liraglutide (ROR = 0.51; 95% CI = 0.38, 0.69; *p* < 0.0001) [27]. Notwithstanding, findings from Shapiro et al. (2025) reported that GLP‐1 RAs were not significantly associated with differences in hazard ratio for suicidal ideations (HR = 0.84; 95% CI = 0.65, 1.08) [19]. These findings suggest that liraglutide may be associated with increased suicidal ideation, especially in comparison to other GLP‐1 RAs.

With respect to the association between semaglutide and suicidal ideation, the extant literature comprised mixed findings. In a retrospective cohort study conducted by Wang et al. (2024) (*n* = 232,771), prescription of semaglutide in patients with obesity was associated with a significantly lowered risk of recurring suicidal ideation when compared to patients with obesity prescribed non–GLP‐1 RA antiobesity medication groups (6.5% vs. 14.1%; hazard ratio [HR] = 0.44; 95% CI = 0.32, 0.60) [22]. Similarly, semaglutide was associated with lower risk of suicidal ideation at 1‐year follow‐up (HR = 0.39, 95% CI = 0.28, 0.53), 2‐year follow‐up (HR = 0.53; 95% CI = 0.41, 0.67), and 3‐year follow‐up (HR = 0.58; 95% CI = 0.49, 0.72) [22]. A separate study by Wadden et al. (2024) also reported that prescription of 2.4 mg semaglutide was associated with reduced instances of suicidal ideation over 68 weeks (0.4% vs. 0.6%) and 104 weeks (0.7% vs. 1.4%) in comparison to placebo based on the C‐SSRS [21].

Separately and in contradistinction, a pharmacovigilance analysis using the EudraVigilance Database (2024) (*n* = 31,444) identified 210 psychiatric adverse events associated with semaglutide prescription, of which suicidal ideation accounted for 40 events (19.0%) [28]. Similarly, a pharmacovigilance analysis by Ruggiero et al. (2024) using the EudraVigilance database (*n* = 41,236) identified 87 adverse events associated with semaglutide prescription that were related to suicide (completed suicide, “depression suicidal,” suicidal behavior, suicidal ideation, suicidal attempt, and suspected suicide), of which suicidal ideations accounted for 67 events (77.9%) [27].

Furthermore, the probability of reporting suicidal events was higher in semaglutide in comparison to dulaglutide (ROR = 2.05; 95% CI = 1.40, 3.01; *p* = 0.0002) and exenatide (ROR = 1.81; 95% CI = 1.08, 3.05; *p* = 0.0229), but lower than that of liraglutide (ROR = 0.51; 95% CI = 0.38, 0.69; *p* < 0.0001) [12]. Similarly, Guirguis et al. (2024) had similar results, wherein semaglutide was associated with increased suicidal ideation (ROR = 1.73; 95% CI = 0.80, 0.30). The aforementioned findings indicate a potential link between semaglutide and suicidal ideation, although further research is required to identify the directionality of this association [17].

#### 3.3.2 Association Between GLP‐1 RAs and Suicidal Attempts

Six studies were identified examining the association between GLP‐1 RAs and suicidal attempts [12, 23, 24, 25, 26, 28]. In a randomized controlled trial by Kelly et al. (2020) (*n* = 251), one death by suicide was recorded following administration of 3.0 mg liraglutide daily over 56 weeks [25]. Notwithstanding, separate investigations deemed the event as unlikely to be related to trial treatment. Two separate pharmacovigilance studies reported no causal link between GLP‐1 RAs and suicidal attempts [12, 28]. Similarly, a pharmacovigilance analysis conducted by Chen et al. (2023) using the FAERS database identified no association between GLP‐1 RAs and increased rates of suicidal attempts with the exception of pediatric populations, wherein an increased rate of suicidal attempts was observed (ROR = 2.50, 95% CI = 1.02, 6.13; *p* = 0.05) [24]. These findings were replicated in a retrospective cohort study by Bezin et al. (2025) (*n* = 6,596), wherein administration of GLP‐1 RAs (i.e., dulaglutide, exenatide, liraglutide, semaglutide) was not associated with increased suicidal behaviors (OR = 0.62; 95% CI = 0.51, 0.75) [23].

In contrast, a retrospective cohort study conducted by Nassar et al. (2024) (*n* = 745,985) reported a significant reduction of risk of suicidal attempts in participants prescribed with GLP‐1 RAs in comparison to dipeptidyl peptidase‐4 inhibitors (DPP‐4is) (28.41 per 100,000 vs. 61.67 per 100,000) with an odds ratio (OR) of 0.461 (95% CI = 0.366, 0.58) [26]. This was mirrored in participants with a history of depression or suicidal attempts, wherein reduced risk of suicidal attempts (76.98 per 100,000 vs. 203.79 per 100,000) was observed [26]. However, a lack of comparison to controls by Nassar et al. (2024) imposed limits on interpreting the relative risk of suicidal attempts associated with GLP‐1 RAs. Taken together, these findings suggest there is no association between GLP‐1 RAs and suicidal attempts.

#### 3.3.3 Association Between GLP‐1 RAs and Suicidal Depression

“Suicidal depression” is a section coded in the FAERS for reports characterizing depression with suicidal thoughts. We identified three studies that evaluated the association between GLP‐1 RAs and suicidal depression [12, 29]. In a randomized controlled trial conducted by Blackman et al. (2016) (*n* = 359), one participant prescribed with liraglutide (1 of 176; 0.6%) reported suicidal depression, although this association was not attributed to liraglutide administration [29]. Although a pharmacovigilance analysis conducted by McIntyre et al. [12] (*n* = 45,415) identified no association between GLP‐1 RA and suicidal depression, a disproportionate reporting of suicidal depression with semaglutide (ROR = 8.81; 95% CI = 3.69, 21.04; *p* ≤ 0.0001) and liraglutide (ROR = 3.74; 95% CI = 1.23, 11.38; *p* ≤ 0.05) was observed [12]. These findings suggest that GLP‐1 RA may have no effect on the risk of suicidal depression. However, further research should be directed to identify the association between suicidal depression with semaglutide and liraglutide.

### 3.4 Association Between Tirzepatide and Suicidality

To examine the association between tirzepatide and suicidality, we examined three studies (Table S3).

#### 3.4.1 Association Between Tirzepatide and Suicidal Ideations

No significant associations between tirzepatide and suicidal ideation were observed when compared to insulin (ROR 1.85; 95% CI = 0.97, 3.51; *p* = 0.06) and metformin (ROR = 1.67; 95% CI = 0.88, 3.15; *p* = 0.12) [12]. This was supported by a randomized controlled trial conducted by Wadden et al. (2023) (*n* = 579), wherein tirzepatide prescription was not significantly associated with suicidal ideation [20]. Contrastingly, Guirguis et al. (2024) reported an association between tirzepatide and increased odds of suicidal ideation (ROR = 1.49; 95% CI = 1.21, −0.41). Evidently, further research is required to examine the possible association between tirzepatide and suicidal ideation [17].

#### 3.4.2 Association Between Tirzepatide and Suicidal Attempts

In a randomized clinical trial by Wadden et al. (2023), no association between tirzepatide prescription and suicide attempts was reported [20]. Additionally, findings by Tobaiqy and Elkout (2024) also reported no association between tirzepatide prescription and suicide attempts [28]. These findings suggest that prescription of tirzepatide may have no effect on suicide attempts, although further research is required to examine this interaction in a larger population.

#### 3.4.3 Association Between Tirzepatide and Suicidal Depression

No reports of suicidality or depression intensification were reported in a randomized controlled trial including participants with obesity using tirzepatide (*n* = 579) [20]. Notwithstanding, further research is evidently required to further examine the aforementioned association.

### 3.5 Association Between GLP‐1 RAs and Depressive Symptoms

To examine the potential mechanisms that subserve changes in suicidality, two included studies also examined the association between GLP‐1 RAs and depressive symptoms [18, 21]. In a post hoc of randomized controlled trials (*n* = 5,325), liraglutide prescription was associated with decreases in mean PHQ‐9 scores following treatment (1.8 ± 2.7 vs. 1.9 ± 2.7), with a treatment difference of −0.02 (95% CI = −0.17, 0.12) [18]. These trends were similarly replicated by Wadden et al. (2024), wherein prescription of semaglutide was also associated with reduced maximum PHQ‐9 scores following 68 weeks (OR = 0.54; 95% CI = 0.37, 0.80; *p* = 0.002), but not following 104 weeks (OR = 0.76; 95% CI = 0.10, 5.80; *p* = 0.79) of semaglutide administration [21]. Evidently, further research is required to examine if GLP‐1 RAs may affect depression and whether this interaction subserves potential associations with suicidality.

## 4 Discussion

In this systematic review, we provided a summary and synthesis of reports documenting an association between GLP‐1 RAs with suicidality (i.e., suicidal ideation and suicidal attempts) and “suicidal depression”. To our knowledge, this is the first systematic review to examine an association between GLP‐1 RAs and domains of suicidality. Notwithstanding the presence of mixed results reported in the extant literature, we identified an association between suicidality and select GLP‐1 RAs. In addition, we also identified a potential differential effect of GLP‐1 RAs on suicidal attempts within pediatric populations.

In our review, we observed an association between liraglutide, semaglutide, and tirzepatide and risk of suicidal ideation across multiple studies. Simultaneously, liraglutide and semaglutide have also been associated with increased reports of “suicidal depression” in the FAERS. However, there was no association between suicidality (i.e., suicidal ideation, attempts and/or “suicidal depression”) and other GLP‐1 RAs including dulaglutide, exenatide, and lixisenatide.

Despite the relationship observed between select GLP‐1 RAs and suicidality, causation cannot be established [2]. As such, no causal link between GLP‐1 RAs and suicidality can be established when using the Bradford Hill criteria [30, 31]. Additionally, recent meta‐analyses by Ebahimi et al. (2025) and Kamrul‐Hasan et al. (2025) reported that GLP‐1 RA and tirzepatide prescription were not associated with increased risk of suicidal ideations and suicide attempts [32, 33].

Notwithstanding, extant evidence has highlighted concerns regarding the potential risk of adverse effects associated with incretin mimetics such as GLP‐1 RAs [34, 35]. In addition, misuse and overdose of these drugs observed in participants in pursuit of weight loss can amplify adverse effects [34, 36]. Taken together, these factors may subserve mechanisms in which GLP‐1 RAs can affect suicidality.

Inferences and interpretations of our systematic review are affected by various methodological limitations. First, our review included participants with different diagnoses (e.g., T2DM, obesity), which may confound interpretation of results. Specifically, obesity has been associated with increased prevalence of depression, which may modulate and subsequently affect baseline incidence rate of the various aspects of suicidality [37]. In conjunction, a relatively small number of studies examined the association between GLP‐1 RAs and suicidality. Notably, pharmacovigilance analyses and clinical trials were used to evaluate the association between GLP‐1 RAs and suicidality. However, due to the nature of pharmacovigilance studies, causal relationships cannot be established. Although randomized controlled trials provide strong evidence with regard to potential causal effects of medications, the relatively low sample size and exclusion criteria (i.e., individuals with depression) may limit the generalizability of their findings. Furthermore, it is important to note that dose‐dependent effects of GLP‐1 RA administration (i.e., liraglutide 1.2 vs. 3.0 mg) are often omitted from the findings of pharmacovigilance studies included in our study. Notably, administration of different doses of liraglutide and various GLP‐1 RAs are intended for different patients (i.e., individuals with diabetes or obesity), wherein differences in patient population may also further confound results. Finally, our findings included studies that examine aspects of suicidality via adverse effect reporting and studies that examine suicidality via established assessments such as the C‐SSRS. Whereas randomized controlled trials typically employ defined, graded scales of suicidal ideation and behavior (i.e., C‐SSRS), pharmacovigilance studies report on adverse events, which rely on judgment by various clinicians and limit the interobserver reliability of these studies. Notwithstanding, clinical trials with larger sample sizes should be conducted to identify the association between disparate GLP‐1 RAs and suicidality. Additionally, the association between GLP‐1 RAs and suicidal attempts in pediatric populations should also be explored.

## 5 Conclusion

Herein, we report an association among liraglutide, semaglutide, and tirzepatide with suicidality. However, a causal relationship between GLP‐1 RAs and suicidality could not be established. These findings provide an updated summary and synthesis on the association between GLP‐1 RAs and suicidality and considerations for future research objectives.

## Funding

The authors have nothing to report.

## Conflicts of Interest

Dr. Roger S. McIntyre has received research grant support from CIHR/GACD/National Natural Science Foundation of China (NSFC), and the Milken Institute; and speaker/consultation fees from Lundbeck, Janssen, Alkermes, Neumora Therapeutics, Boehringer Ingelheim, Sage, Biogen, Mitsubishi Tanabe, Purdue, Pfizer, Otsuka, Takeda, Neurocrine, Neurawell, Sunovion, Bausch Health, Axsome, Novo Nordisk, Kris, Sanofi, Eisai, Intra‐Cellular, NewBridge Pharmaceuticals, Viatris, Abbvie, and Atai Life Sciences. Kayla M. Teopiz has received fees from Braxia Scientific Corp. Dr. Joshua D. Rosenblat has received research grant support from the Canadian Institute of Health Research (CIHR), Physician Services Inc. (PSI) Foundation, Labatt Brain Health Network, Brain and Cognition Discovery Foundation (BCDF), Canadian Cancer Society, Canadian Psychiatric Association, Academic Scholars Award, American Psychiatric Association, American Society of Psychopharmacology, University of Toronto, University Health Network Centre for Mental Health, Joseph M. West Family Memorial Fund, and Timeposters Fellowship and industry funding for speaker/consultation/research fees from iGan, Boehringer Ingelheim, Janssen, Allergan, Lundbeck, Sunovion, and COMPASS. Hezekiah C. T. Au, Yang Jing Zheng, Gia Han Le, Sabrina Wong, Angela T. H. Kwan, and Hayun Choi declare no conflicts of interest.

## Supporting information


**Table S1:** Risk of bias/quality assessment of the included studies using the NIH Quality Assessment Tool of Controlled Intervention Studies (Ma et al., 2020; NIH, 2013).
**Table S2:** Risk of bias/quality assessment of the included studies using the NIH Quality Assessment Tool for Observational Cohort and Cross‐Sectional Studies (Ma et al., 2020; NIH, 2013).
**Table S3:** Summary of trends observed in the studies examining the association between GLP‐1 RAs and suicidality.

## Data Availability

Data sharing not applicable to this article as no datasets were generated or analysed during the current study.
