# Supplementary material for: Association of Glucagon‐Like Peptide‐1 Receptor Agonists and Suicidality: A Systematic Review
Source: Obes Rev. 2026 Mar 6;27(8):e70120. doi: 10.1111/obr.70120 (PMC13371786; doi:10.1111/obr.70120)
Supplement: Supplementary file 1 — Table S1: Risk of bias/quality assessment of the included studies using the NIH Quality Assessment Tool of Controlled Intervention Studies (Ma et al., 2020; NIH, 2013). Table S2: Risk of bias/quality assessment of the included studies using the NIH Quality Assessment Tool for Observational Cohort and Cross‐Sectional Studies (Ma et al., 2020; NIH, 2013). Table S3: Summary of trends observed in the studies examining the association between GLP‐1 RAs and suicidality. [file OBR-27-e70120-s001.pdf]

Association of Glucagon-Like Peptide-1 Receptor Agonists and Suicidality: A Systematic Review

Hezekiah C.T. Au[1,2,3], hezekiah.au@mail.utoronto.ca

Yang Jing Zheng[2,3], yangjing.zheng@mail.utoronto.ca

Gia Han Le, HBSc[2,3,4], hanny.le@mail.utoronto.ca

Sabrina Wong, HBSc[3,4,5], sabrinal.wong@mail.utoronto.ca

Kayla M. Teopiz, HBSc[2,3], kayla.teopiz@mail.utoronto.ca

Angela T.H. Kwan, MSc[3,6], angela.kwan@mail.utoronto.ca

Joshua D. Rosenblat, MD, FRCPC[3,4,7], joshua.rosenblat@uhn.ca

Rodrigo B. Mansur, MD, PhD[3,4,7], rodrigo.mansur@uhn.ca

Hayun Choi, MD[3,7,8], hayun.choi@utoronto.ca

Roger S. McIntyre, MD, FRCPC[7]*, roger.mcintyre@bcdf.org

Affiliations

1. Joint Department of Medical Imaging, University of Toronto, Toronto, Ontario, Canada
2. Institute of Medical Science, University of Toronto, Toronto, Ontario, Canada
3. Brain and Cognition Discovery Foundation, Toronto, Ontario, Canada
4. Mood Disorder Psychopharmacology Unit, University Health Network, Toronto, Ontario, Canada
5. Department of Pharmacology and Toxicology, University of Toronto, Toronto, Ontario, Canada
6. Faculty of Medicine, University of Ottawa, Ottawa, Ontario, Canada
7. Department of Psychiatry, University of Toronto, Ontario, Canada
8. Department of Psychiatry, Veteran Health Service Medical Center, Seoul, Republic of Korea

*Corresponding Author: Dr. Roger S. McIntyre, Brain and Cognition Discovery Foundation, 77 Bloor Street West, Suite 617, Toronto, ON, M5S 1M2, Canada

SUPPLEMENTARY MATERIALS

Table S1. Risk of bias/quality assessment of the included studies using the NIH Quality Assessment Tool of Controlled Intervention Studies (Ma et al., 2020; NIH, 2013).

Study	Item														Quality Rating
	1	2	3	4	5	6	7	8	9	10	11	12	13	14	
Blackman et al. (2016)	✓	✓	✓	✓	✓	✓	X	✓	✓	✓	✓	✓	✓	✓	Good
Kelly et al. (2020)	✓	X	X	✓	✓	✓	✓	✓	✓	✓	✓	✓	✓	✓	Good
O'Neil et al. (2017)	✓	✓	✓	✓	✓	✓	NR	NR	✓	✓	✓	X	X	✓	Good
Wadden et al. (2023)	✓	X	X	✓	✓	NR	✓	✓	✓	✓	✓	✓	✓	✓	Good
Wadden et al. (2024)	✓	✓	✓	✓	✓	✓	NR	NR	NR	✓	✓	X	✓	✓	Good

Symbols: ✓ - yes; X - no

Abbreviations: NR = not reported; NA = not applicable; CD = cannot determine

Table S2. Risk of bias/quality assessment of the included studies using the NIH Quality Assessment Tool for Observational Cohort and Cross-Sectional Studies (Ma et al., 2020; NIH, 2013).

Study	Item														Quality Rating
	1	2	3	4	5	6	7	8	9	10	11	12	13	14	
Bezin et al. (2025)	✓	✓	NA	X	X	✓	✓	NA	✓	X	✓	X	NA	✓	Good
Nassar et al. (2024)	✓	✓	NA	X	X	✓	✓	✓	✓	X	✓	X	NA	X	Good
Shapiro et al. (2025)	✓	✓	NA	X	X	✓	✓	NA	✓	X	✓	X	NA	✓	Good
Wang et al. (2023)	✓	✓	NA	X	X	✓	✓	NA	✓	✓	✓	X	NA	X	Good

Symbols: ✓ - yes; X - no

Abbreviations: NR = not reported; NA = not applicable; CD = cannot determine

Table S3. Summary of trends observed in the studies examining the association between GLP-1 RAs and suicidality

Study	Suicidal Behavior		
	**Suicidal Ideation**	**Suicidal Attempt**	**Suicidal Depression**
Bezin et al. (2025)		**Dulaglutide, Exenatide, Liraglutide, Semaglutide** Reduced	
Blackman et al. (2016)			**Liraglutide** None

Chen et al. (2023)		Increased in children	
Guirguis et al. (2024)	**Semaglutide, Liraglutide, Tirzepatide**<br>Increased		
Kelly et al. (2020)		**Liraglutide**<br>None	
McIntyre et al. (2024)	**Semaglutide, Liraglutide**<br>Increased<br><br>**Dulaglutide, Exenatide, Lixisenatide, Tirzepatide**<br>None	None	**Semaglutide, Liraglutide**<br>Increased<br><br>**Dulaglutide, Exenatide, Lixisenatide, Tirzepatide**<br>None
Nassar et al. (2024)		Reduced	
O'Neil et al. (2017)	**Liraglutide**<br>None		
Ruggiero et al. (2024)	**Semaglutide, Liraglutide**<br>Increased		
Shapiro et al. (2025)	None		
Tobaiqy and Elkout (2024)	**Semaglutide, Liraglutide**<br>Reduced	**Semaglutide, Liraglutide, Tirzepatide**	

	**Tirzepatide**<br>None	None	
Wadden et al. (2023)	**Tirzepatide**<br>None	**Tirzepatide**<br>None	**Tirzepatide**<br>None
Wang et al. (2024)	**Semaglutide**<br>Reduced risk		
Wadden et al. (2024)	**Semaglutide**<br>Reduced		
